# Potent cytotoxicity and induction of ROS-mediated genomic instability, mitochondrial dysfunction, and apoptosis by Y_2_O_3_ NPs in Hep-G2 hepatic cancer cells

**DOI:** 10.1007/s00210-025-04051-9

**Published:** 2025-04-10

**Authors:** Hanan R. H. Mohamed, Rawan Essam, Basma A. Mohamed, George M. Hakeem, Shahd H. Elnawasani, Maria Nagy, Gehan Safwat, Ayman Diab

**Affiliations:** 1 Department of Zoology, Faculty of Science, Cairo University, Giza, Egypt; 2 Faculty of Biotechnology, October University for Modern Sciences and Arts (MSA), 6Th of October City, Egypt

**Keywords:** Yttrium oxide nanoparticles, Hepatic cancer, Genomic Instability, Mitochondrial dysfunction and apoptosis induction

## Abstract

Hepatic cancer, one of the most prevalent and lethal cancers globally, remains a significant health challenge, with limited treatment options underscoring the urgent need for novel, more effective therapies. Yttrium oxide nanoparticles (Y_2_O_3_ NPs) have attracted attention in nanomedicine due to their promising properties, including enhanced drug delivery, imaging capabilities, and therapeutic effects. However, the specific impact of Y_2_O_3_ NPs on hepatic cancer is largely unexplored. Therefore, this study was conducted to assess the cytotoxic effects of Y_2_O_3_ NPs on cell viability, reactive oxygen species (ROS) generation, genomic stability, mitochondrial integrity, and apoptosis induction in Hep-G2 hepatic cancer cells. The results from the SRB cytotoxicity assay demonstrated a strong concentration-dependent decrease in Hep-G2 cell viability, with a notably low half-maximal inhibitory concentration (IC50) value of 13.15 µg/ml. Exposure to the IC50 concentration of Y_2_O_3_ NPs led to increased ROS generation, DNA damage induction, and loss of mitochondrial membrane potential. Furthermore, the expression of pro-apoptotic p53 and mitochondrial ND3 genes was significantly upregulated, while the anti-apoptotic Bcl-2 gene was markedly downregulated, triggering apoptosis in Hep-G2 cells after 72 h of exposure to Y_2_O_3_ NPs. Collectively, these findings highlight the therapeutic potential of Y_2_O_3_ NPs in hepatic cancer, emphasizing the need for further research to fully explore their efficacy as a treatment option for liver cancer.

## Introduction

Hepatocellular carcinoma (HCC) is the most prevalent form of liver cancer, making up about 75–85% of all liver cancer cases globally. It represents a major global health issue, with approximately 900,000 new cases and 830,000 deaths each year, positioning it as the third leading cause of cancer-related deaths worldwide (Forner et al. 2018; Villanueva 2019). The incidence of HCC varies by region, with the highest rates seen in East Asia and sub-Saharan Africa due to the widespread prevalence of chronic hepatitis B virus (HBV) and hepatitis C virus (HCV) infections, which are major risk factors. Other key risk factors include alcohol-induced liver disease, non-alcoholic fatty liver disease (NAFLD), and exposure to aflatoxins, particularly in endemic regions (Llovet et al. 2021; Sung et al. 2021).

The progression to HCC typically occurs in the context of chronic liver disease and cirrhosis, underscoring the importance of monitoring high-risk populations (Llovet et al. 2021). Hepatic cancer, particularly HCC, is one of the leading causes of cancer-related deaths worldwide. Despite advances in cancer treatment, the therapeutic options for HCC remain limited. Surgical resection and liver transplantation offer the best chances for long-term survival, but these are only viable for patients with early-stage disease. For those with advanced-stage HCC, available treatments, including chemotherapy, radiation therapy, and targeted therapies often provide limited efficacy and are associated with significant side effects. However, systemic therapies, including tyrosine kinase inhibitors like sorafenib and lenvatinib, as well as immune checkpoint inhibitors such as atezolizumab in combination with bevacizumab, have shown promising results in improving survival outcomes (Finn et al. 2020; Cheng et al. 2022).

Chemotherapy is a crucial treatment option for many cancers, but it comes with a range of potential side effects, particularly for patients with hepatic cancer. These side effects arise because chemotherapy drugs target and kill rapidly dividing cancer cells, but they can also damage healthy cells that divide quickly, leading to systemic toxicity. Common side effects include nausea, vomiting, fatigue, hair loss, myelosuppression, gastrointestinal disturbances, cardiotoxicity, nephrotoxicity, and even hepatotoxicity. These adverse toxic effects can significantly reduce a patient's quality of life and may limit the dose and duration of treatment (Vogel et al. 2021; Wu et al. 2017; Li et al. 2019; Shiji et al. 2022; Shamjith et al. 2022). Consequently, there is a an urgent need for alternative therapies that are not only effective but also offer fewer or more manageable side effects, particularly for patients with liver cancer who may already have compromised liver function.

Nanotechnology-based therapies are emerging as promising alternatives for cancer treatment, offering targeted drug delivery, improved therapeutic efficacy, and reduced systemic toxicity (Yao et al. 2020). Among various nanomaterials, Yttrium oxide nanoparticles (Y_2_O_3_ NPs) stand out due to their unique properties, including high stability, biocompatibility, and strong luminescent behavior. These characteristics make Y₂O₃ NPs highly suitable for biomedical applications, particularly in hepatic cancer therapy, where they can facilitate targeted treatment, improve imaging for diagnosis, and induce selective cytotoxicity in cancer cells. Their ability to generate reactive oxygen species (ROS) further enhances their therapeutic potential by promoting apoptosis in malignant cells (Akhtar et al. 2020). Despite these promising characteristics, there is a notable lack of research on the effects of Y_2_O_3_ NPs in hepatic cancer. Most research on liver cancer nanomedicine has focused on other nanoparticles, such as gold, iron oxide, and silica-based materials, which have been more extensively studied for their interactions with liver cancer cells (Taghizadeh et al. 2019; Liu et al. 2021; Escutia-Gutiérrez et al. 2023).

Although research on Y_2_O_3_ NPs in hepatic cancer is limited, their promising properties make them an important area for further exploration. Recent studies have demonstrated the strong anti-cancer effects of Y_2_O_3_ NPs against breast and epidermoid skin cancers, with no toxicity to normal cells (Emad et al. 2023; Mohamed et al. 2025). Y_2_O_3_ NPs have been shown to induce significant reactive oxygen species (ROS) production in human triple-negative MDA-MB-231 breast cancer cells, causing severe DNA damage, apoptosis, and ferroptosis. Similarly, Y_2_O_3_ NPs induced high level of ROS in human epidermoid A431 skin cancer cells, leading to dramatic genomic and mitochondrial DNA damage, loss of mitochondrial membrane potential, and changes in the expression of apoptotic and mitochondrial genes, triggering apoptosis (Emad et al. 2023; Mohamed et al. 2025). On the contrary, Y_2_O_3_ NPs were non-toxic to human normal retina RPE1, dermal fibroblasts HDF and human skin fibroblast HSF cells (Emad et al. 2023; Mohamed et al. 2025).

This discovery presents exciting potential for cancer research, as the selective effects of Y_2_O_3_ NPs on cancer cells versus normal cells suggest their potential use in targeted cancer therapies. The ability of Y_2_O_3_ NPs to induce apoptosis and ferroptosis in cancer cells while sparing normal cells is a significant advantage, potentially leading to more effective and less toxic treatments (Emad et al. 2023; Mohamed et al. 2025). However, there is still limited research on their impact on hepatic cancer cells, highlighting the need for further investigation into their role in liver cancer therapy. Therefore, the current study was designed to assess the effects of Y_2_O_3_ NPs on cell viability, genomic and mitochondrial DNA integrity, reactive oxygen species generation, mitochondrial membrane potential, and apoptosis induction in hepatocellular carcinoma Hep-G2 cells.

## Materials and methods

### Chemicals

Powders of Y_2_O_3_ NPs were acquired from Sigma-Aldrich Company (St. Louis, MO, USA), with a product number of 544892. The Y_2_O_3_-NPs has an average particle size of less than 50 nm, and a purity of 99.9% trace metals. All other materials and supplies utilized in the experiments throughout this study were sourced at high molecular grade to ensure consistency and accuracy in the experimental procedures.

### Characterization of Y_2_O_3_ NPs

Y_2_O_3_ NPs utilized in this study have been thoroughly characterized by Emad et al., (2023) and Mohamed et al*. *(2025). The purity of the purchased Y_2_O_3_ NPs powders was confirmed through X-ray diffraction analysis, while the stability and uniform dispersion of the suspended Y_2_O_3_ NPs were assessed using dynamic light scattering. Transmission electron microscopy (TEM) imaging revealed that the Y_2_O_3_ NPs had a spherical shape and an average particle size of 14.00 nm (Emad et al. 2023; Mohamed et al. 2025).

### Cell culture

Human hepatocellular carcinoma Hep-G2 cells were obtained from Nawah Scientific Inc. (Mokatam, Cairo, Egypt). The Hep-G2 cells were cultured in Dulbecco's Modified Eagle Medium (DMEM) supplemented with 10% inactivated fetal bovine serum, 100 units/ml penicillin, and 100 mg/ml streptomycin. The cells were kept in an incubator at 37 °C with 5% CO2 to provide optimal growth conditions.

### Estimation of Y_2_O_3_ NPs cytotoxicity

The influence of Y_2_O_3_ NPs on the viability of hepatic Hep-G2 cancer cells was assessed using a Sulforhodamine B (SRB) cytotoxicity assay (Skehan et al. 1990; Allam et al. 2018). Hep-G2 cells (100 µl suspension) were seeded in 96-well plates and incubated in complete media for 24 h. The cells were then exposed to five different concentrations of Y_2_O_3_ NPs (0.2, 2, 20, 200, and 2000 µg/ml) for 72 h. Following treatment, the cells were fixed, washed with distilled water, and incubated with SRB solution (0.4%) for 10 min in the dark at room temperature. The plates were then washed with 1% acetic acid and left to dry overnight. The SRB bound to the proteins was dissolved, and absorbance was measured at 540 nm using a BMG LABTECH®-FLUO Star Omega microplate reader (Ortenberg, Germany). The half-maximal inhibitory concentration (IC50) of Y_2_O_3_ NPs in Hep-G2 cells was calculated from triplicate measurements using GraphPad Prism software.

### Experimental design

Hepatic Hep-G2 cancer cells were cultured in T25 flasks under optimal conditions. Once the cells reached appropriate confluence, they were divided into two distinct groups: untreated (control) and Y_2_O_3_ NPs-treated cells. The control group was exposed to a vehicle solution of dimethyl sulfoxide (DMSO) at a concentration of less than 0.1%, while the experimental group was treated with Y_2_O_3_ NPs at the IC50 concentration for a duration of 72 h.

After treatment, both the control and treated cells underwent the following steps: first, they were harvested by centrifugation, followed by trypsinization to detach the cells from the flask surface. The cells were then washed twice with ice-cold phosphate-buffered saline (PBS) to remove any residual treatment medium. The final cell pellets were stored at −80 °C in PBS for future molecular analysis, including various assays to assess cell viability, gene expression, and other cellular responses. To ensure the accuracy and reliability of the results, the Y_2_O_3_ NPs treatment was conducted in triplicates. This approach was designed to minimize experimental variability and provide robust data for statistical analysis, ensuring the precision and consistency of the findings.

### Estimation of Y_2_O_3_ NPs induced DNA damage

Level of genomic DNA damage in untreated and Y_2_O_3_ NPs-treated Hep-G2 cells was measured using the single-cell gel electrophoresis (alkaline comet) assay (Tice et al. 2000; Langie et al. 2015). A 15 µl suspension of Hep-G2 cells was gently mixed with 60 µl of low-melting agarose and spread evenly onto a slide pre-coated with normal-melting agarose (1%), then left to solidify. The slides were incubated in cold lysis buffer with freshly added DMSO and Triton-X100, in the dark, for 24 h at 4 °C. After lysis, the slides were immersed in freshly prepared alkaline electrophoresis buffer (pH > 12) for 15 min and then electrophoresed for 30 min at 25 V and 300 mA. Following electrophoresis, the DNA was neutralized, fixed, dried, and stained with ethidium bromide for imaging. The resulting comet nuclei, reflecting various levels of DNA damage, were analyzed using COMETSCORE™ software. Comet parameters, including tail length, %DNA in the tail, and tail moment, were reported as mean ± standard deviation (SD).

### Estimation of ROS generation level

The level of ROS generation in untreated and Y_2_O_3_ NPs-treated hepatic Hep-G2 cancer cells was assessed using 2,7-dichlorofluorescin diacetate (2,7-DCFH-DA) dye (Siddiqui et al. 2010). An equal volume of Hep-G2 cell suspension and 2,7-DCFH-DA dye (20 mM) was gently mixed and left in darkness for 30 min at room temperature. During this incubation, the 2,7-DCFH-DA dye passively entered the cells and specifically reacted with ROS forming the fluorescent compound dichlorofluorescein. After incubation, the mixture was spread evenly on a clean slide and examined under an epi-fluorescent microscope at 200X magnification. Images were captured, and the emitted fluorescent light was analyzed to indicate ROS production in the Hep-G2 cancer cells.

### Estimation of the mitochondrial membrane potential integrity

The impact of Y_2_O_3_ NPs on the mitochondrial membrane potential of hepatic Hep-G2 cancer cells was estimated using the fluorescent dye Rhodamine-123 (Zhang et al. 2011). An equal volume of Hep-G2 cell suspension and Rhodamine-123 dye (10 mg/ml) was gently mixed and incubated for 1 h at 37 °C in the dark. After incubation, the cells were washed twice with PBS, evenly spread on a clean sterile slide, and examined under an epi-fluorescence microscope at 200 × magnification. The emitted fluorescence from the Rhodamine-123 stained cells was then analyzed.

### Estimation of apoptosis induction

Apoptosis in untreated and Y_2_O_3_ NPs-treated Hep-G2 hepatic cancer cells was detected using Flow Cytometry using the Annexin V-Fluorescein isothiocyanate (FITC) apoptosis detection kit (Abcam Inc., Cambridge, UK). A dual-channel Flow Cytometry was utilized to discriminate between apoptotic and necrotic cells. After treating Hep-G2 cells with Y_2_O_3_ NPs for 72 h, the cells were collected via trypsinization and washed twice with ice-cold PBS (pH 7.4). The collected cells were incubated in darkness with an Annexin V-FITC/propidium iodide (PI) solution for 30 min at room temperature. The cells were then analyzed using the ACEA Novocyte Flow Cytometer (ACEA Biosciences Inc., San Diego, CA, USA), with FITC and PI fluorescence measured by FL1 and FL2 detectors, respectively (λex/em 488/530 nm for FITC and λex/em 535/617 nm for PI). A total of 12,000 events were acquired per sample, and positive FITC and/or PI cells were quantified through quadrant analysis using ACEA NovoExpress software (ACEA Biosciences Inc., San Diego, CA, USA).

### Quantifying gene expression

Quantitative Real-Time Polymerase Chain Reaction (qRT-PCR) was done to assess the expression level of the apoptotic p53 gene, the anti-apoptotic Bcl2 gene, and the mitochondrial ND3 gene in Hep-G2 hepatic cancer cells. Total RNA was extracted from Hep-G2 cells according to the manufacturer's instructions using Thermo Fisher Scientific's GeneJET RNA Purification Kit (USA). One microgram of the extracted RNA was reverse-transcribed into complementary DNA (cDNA) using the cDNA Reverse Transcription Kit from Applied Biosystems (Foster City, CA, USA). A qRT-PCR was then performed for each sample separately using the StepOnePlus Real-Time PCR System (Applied Biosystems), with SYBR Green PCR Master Mix and the primer sequences outlined in Table 1, as previously designed by Suzuki et al. (1999), Lai et al. (2012), and Grzybowska-Szatkowska et al. (2014). The expression level of the p53, Bcl2, and ND3 genes were standardized against the housekeeping gene GAPDH, and the fold change in gene expression was calculated using the comparative Ct (ΔΔCt) method. The results are presented as mean ± SD.

**Table 1:** Sequences of primers used in qRT-PCR

Gene	Strand	Primer's sequences
GAPDH	Forward	5'-GAAGGTGAAGGTCGGAGTCA-3'
	Reverse	5'-GAAGATGGTGATGGGATTTC-3'
ND3	Forward	5'-CGCCGCCTGATACTGGCAT-3’
	Reverse	5'-CTAGTATTCCTAGAAGTGAG-3'
BCL-2	Forward	5'-TCCGATCAGGAAGGCTAGAGT-3'
	Reverse	5'-TCGGTCTCCTAAAAGCAGGC-3'
P53	Forward	5'-CAGCCAAGTCTGTGACTTGCACGTAC-3'
	Reverse	5'-CTATGTCGAAAAGTGTTTCTGTCATC-3'

### Statistical analysis

**Fig. 1:**
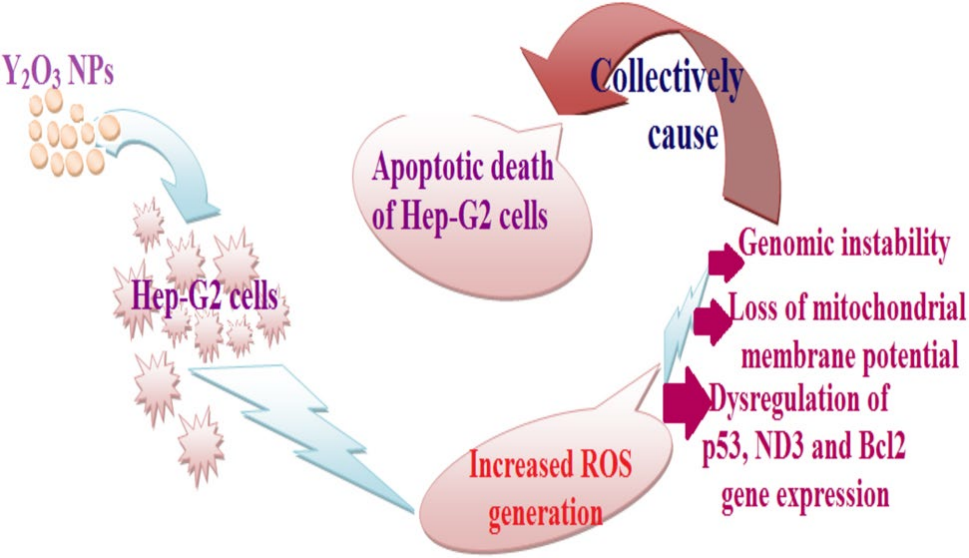
A detailed schematic shows an overview of the effect of Y_2_O_3_ NPs exposure on hepatic Hep-G2 cancer cells

All results were analyzed using the Statistical Package for the Social Sciences (SPSS) and are presented as mean ± SD. An unpaired Student's t-test was employed to compare the untreated and Y_2_O_3_ NPs-treated cells. A detailed schematic diagram summarize the overall effect of Y_2_O_3_ NPs on hepatic Hep-G2 cancer cells has been displayed in Fig. 1.

## Results

### Y_2_O_3_ NPs induce potent cytotoxicity

**Fig. 2:**
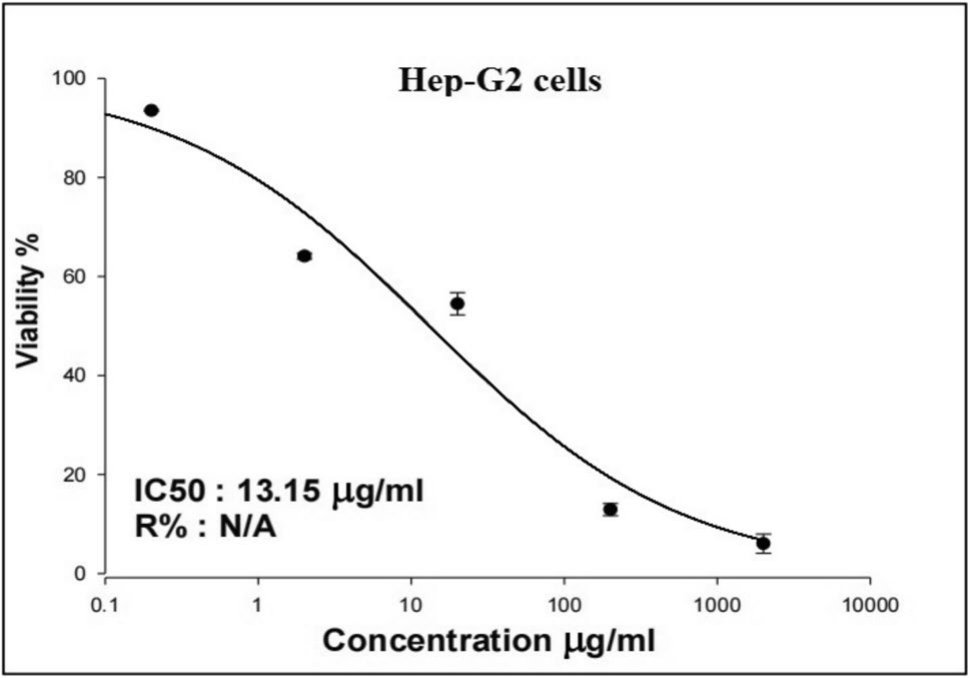
Viability of hepatic Hep-G2 cancer cells following exposure to five different concentrations (0.2, 2, 20, 200 and 2000 µg/ml) of Y_2_O_3_ NPs for 72 h. Triplicate was conducted for each concentration

The results of SRB cytotoxicity assay demonstrated the strong cytotoxic effect of Y_2_O_3_ NPs on hepatic Hep-G2 cancer cells, as manifested by the remarkable concentration-dependent decrease in cell viability observed after exposure to Y_2_O_3_ NPs at concentrations of 0.2, 2, 20, 200, and 2000 µg/ml (Fig. 2). This strong cytotoxicity of Y_2_O_3_ NPs was further confirmed by the found low IC50 value of 13.15 µg/ml (Fig. 2).

### Y_2_O_3_ NPs cause severe DNA damage

**Fig. 3:**
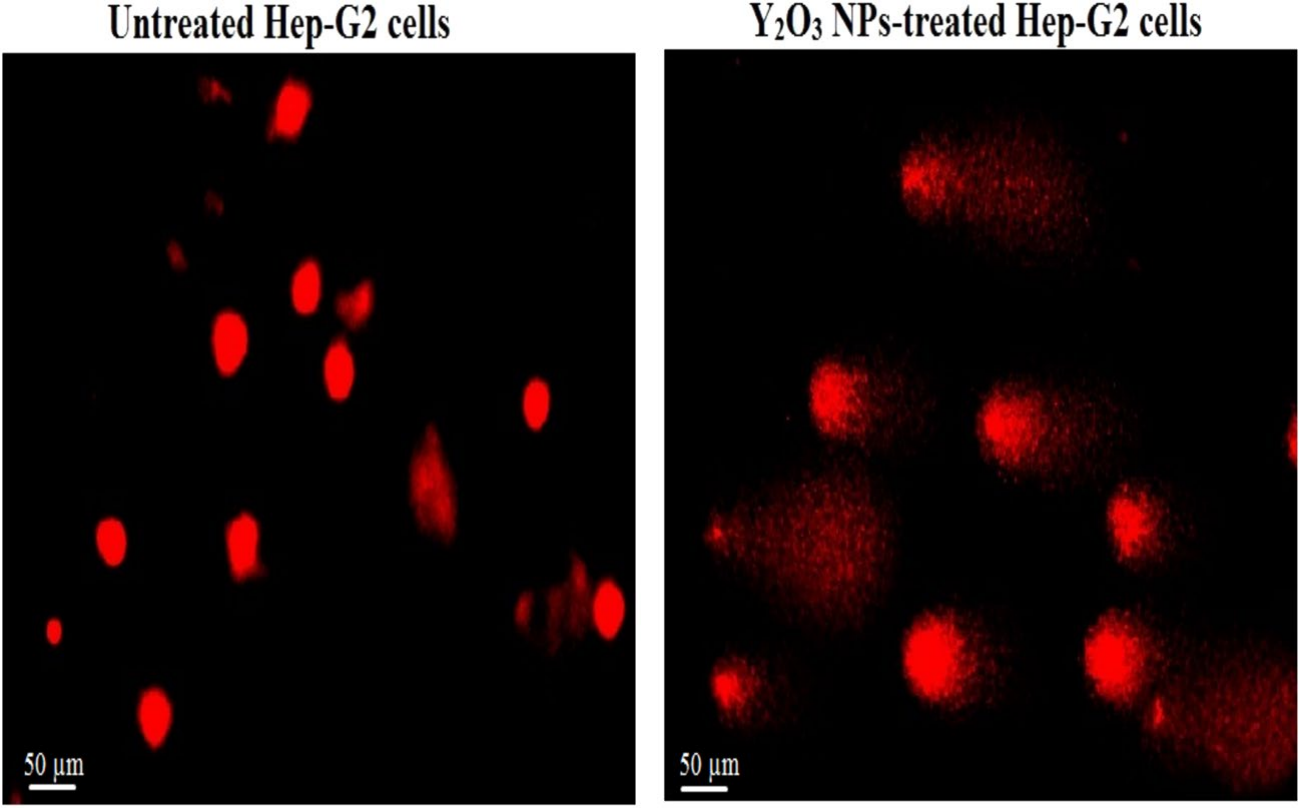
Comet nuclei of untreated Hep-G2 cells with intact DNA and Comet nuclei of Hep-G2 cells treated with IC50 concentration of Y_2_O_3_ NPs for 72 h showing various DNA damage degrees. Magnification 200x

As shown in Table 2 and Fig. 3, exposure of hepatic Hep-G2 hepatic cancer cells to Y_2_O_3_ NPs at an IC50 concentration of 13.15 µg/ml for 72 h resulted in dramatic genomic DNA damage. This severe damage was reflected by the statistically significant (p < 0.01) increases seen in DNA damage parameters: tail length, %DNA in the tail, and tail moment in the treated Hep-G2 cells compared to the untreated control cells (Table 2).

**Table 2:** Level of genomic DNA damage in hepatic Hep-G2 cancer cells following exposure to an IC50 concentrations (13.15 µg/ml) of Y_2_O_3_ NPs for 72 h

Cells	Treatment (µg/ml)	DNA damage parameters		
		Tail length (px)	%DNA in tail	tail moment
Hep-G2 cells	Untreated (0.00 µg/ml)	4.89 ± 0.99	22.91 ± 1.60	1.13 ± 0.24
	Y_2_O_3_ NPs-treated (13.15 µg/ml)	11.25 ± 1.85^**^	38.41 ± 3.88^**^	3.93 ± 0.95^**^

Results are expressed as mean ± SD

^** ^Indicates statistical significant difference from the compared untreated control cells at p < 0.01 using *independent student t-test*

### Y_2_O_3_ NPs cause over ROS production

**Fig. 4:**
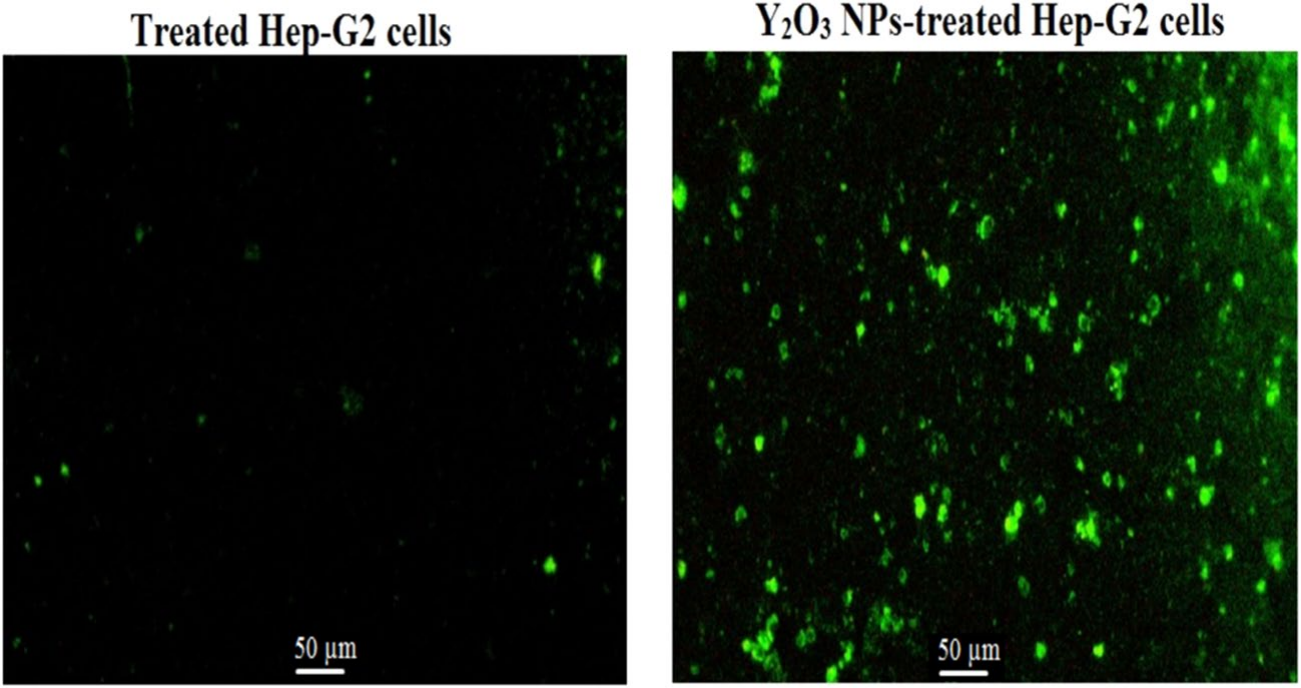
Level of ROS generation within untreated and treated Hep-G2 cancer cells with an IC50 concentration (13.15 µg/ml) of Y_2_O_3_ NPs for 72 h. Magnification 200x

Studying the ROS generation using 2,7-DCFH-DA dye revealed a marked elevation in ROS generation within hepatic Hep-G2 cancer cells exposed to IC50 concentration (13. 15 µg/ml) of Y_2_O_3_ NPs for 72 h (Fig. 4). This increased production was manifested by the remarkable increase in the intensity of fluorescence emitted by Y_2_O_3_ NPs-treated Hep-G2 cells compared to the fluorescence emitted by untreated Hep-G2 cells.

### Y_2_O_3_ NPs cause dramatic loss of mitochondrial membrane potential integrity

**Fig. 5:**
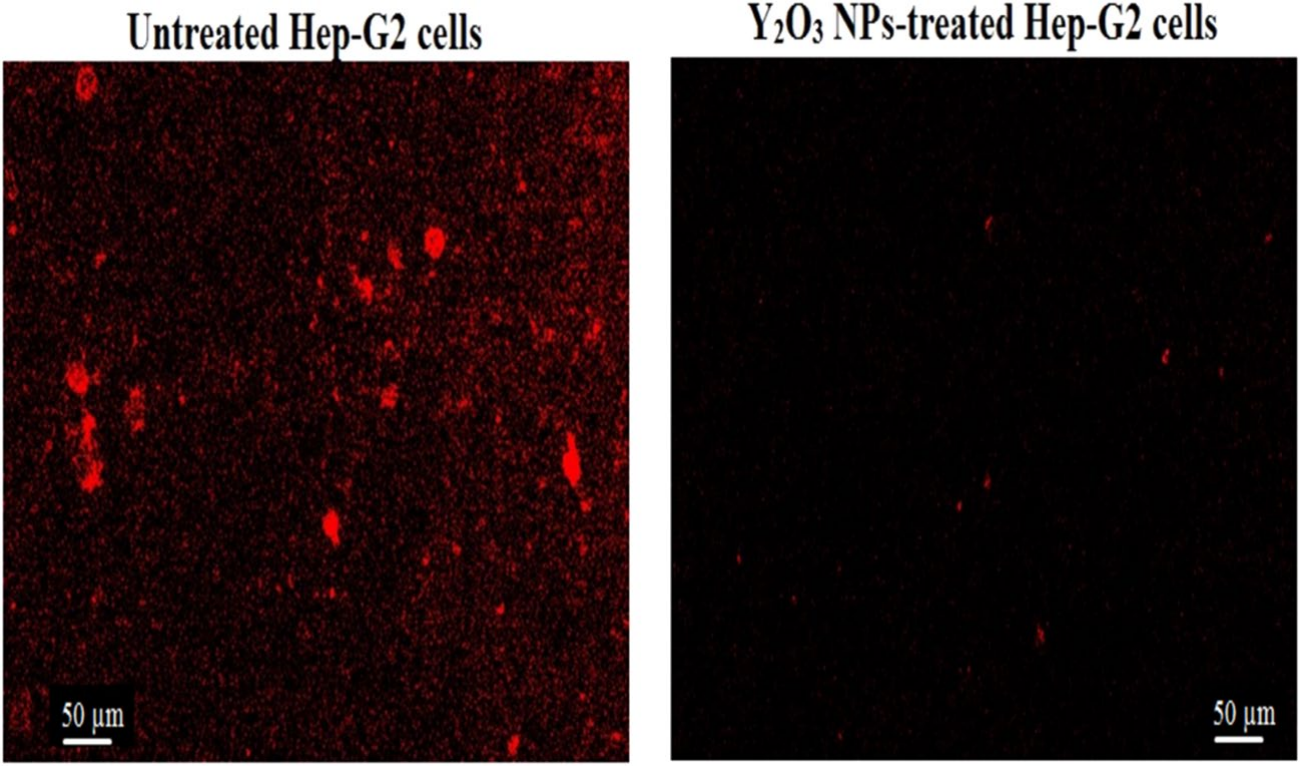
Integrity of mitochondrial membrane potential in the untreated and treated hepatic Hep-G2 cells with an IC50 concentration (13.15 µg/ml) of Y_2_O_3_ NPs for 72 h. Magnification 200x

As noticed in Fig. 5, exposure to Y_2_O_3_ NPs at IC50 concentration (13.15 µg/ml) for 72 h caused a high reduction in the integrity of the mitochondrial membrane potential in hepatic hep-G2 cells. This dramatic loss of mitochondrial membrane potential was revealed by the marked decrease in the intensity of the fluorescence light emitted from Y_2_O_3_ NPs-treated Hep-G2 cells compared to the slightly emitted light from untreated Hep-G2 cells (Fig. 5).

### Y_2_O_3_ NPs induce apoptosis and necrosis of Hep-G2 cells

**Fig. 6:**
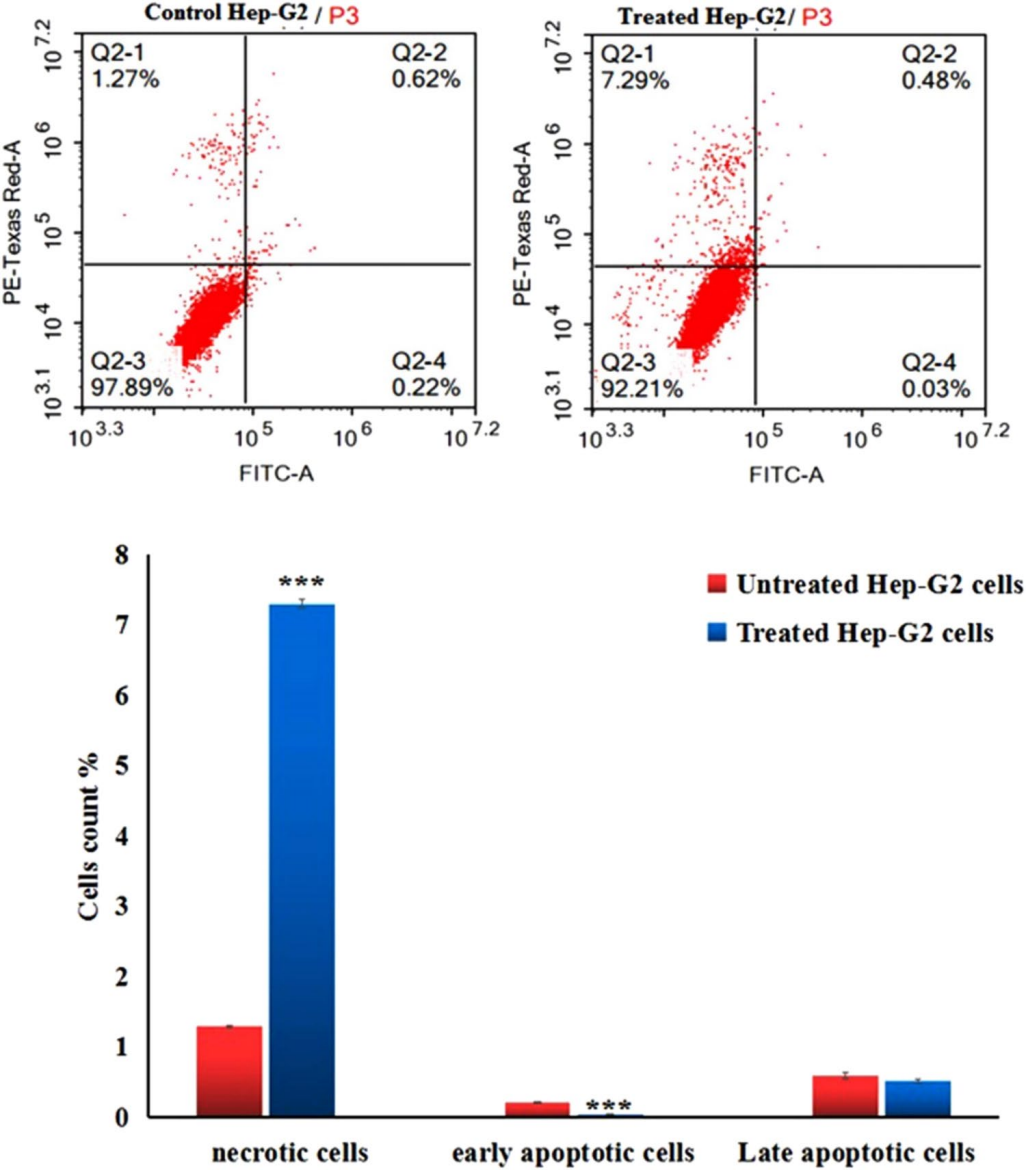
Apoptosis induction in the untreated (control) and treated hepatic Hep-G2 cancer cells with an IC50 concentration (13.15 µg/ml) of Y_2_O_3_ NPs for 72 h. Results are expressed as mean ± SD and analyzed using *independent student t-test*. ***: Indicates statistical significant difference from the compared untreated control cells at p < 0.01 and 0.001. Q2-1 denotes necrosis phase; Q2-2 denotes late apoptosis phase, Q2-3 denotes normal viable cells and Q2-4 denotes early apoptosis phase

Results of Flow cytometry demonstrated apoptosis and necrosis of hepatic Hep-G2 cancer cells upon exposure to IC50 concentration (13.15 µg/ml) of Y_2_O_3_ NPs for 72 h (Fig. 6). Apoptosis and necrosis of Y_2_O_3_ NPs-treated Hep-G2 cells were manifested by the statistically significant (P < 0.001) elevations in the number of early apoptotic and necrotic Hep-G2 cells compared with the corresponding number of untreated early apoptotic and necrotic hep-G2 cells (Fig. 6).

### Y_2_O_3_ NPs markedly dysregulate p53, ND3 and Bcl2 gene expression

**Fig. 7:**
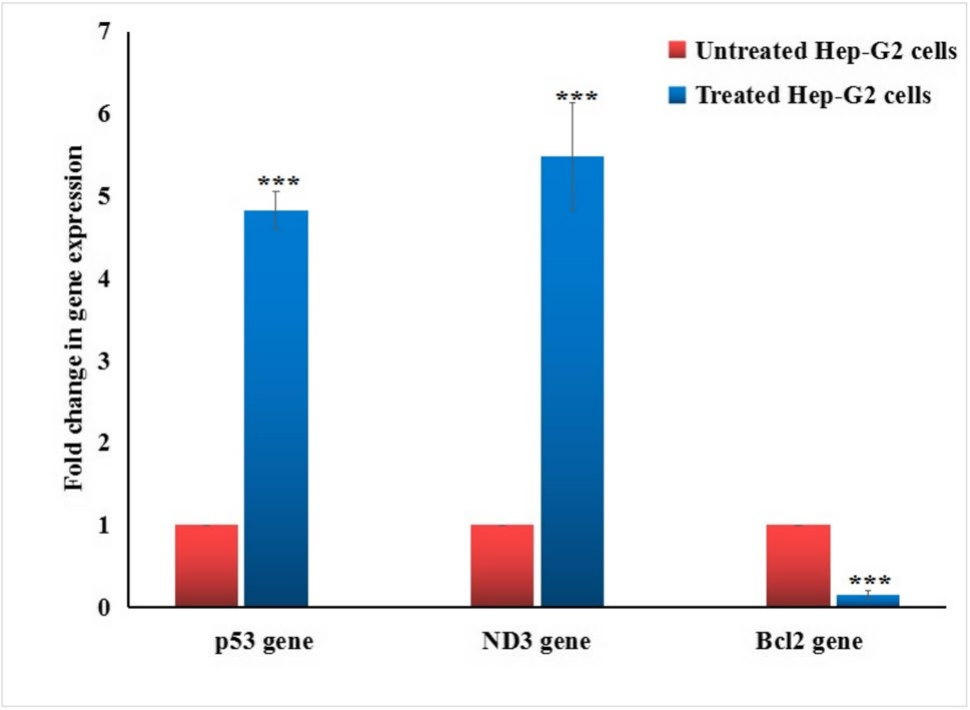
Expression level of p53, ND3 and Bcl2 genes in hepatic Hep-G2 cancer cells following exposure to an IC50 concentration (13.15 µg/ml) of Y_2_O_3_ NPs for 72 h. Results are expressed as mean ± SD and analyzed using *independent student t-test*. ***: Indicates statistical significant difference from the compared untreated control cells at p < 0.01 and 0.001

As seen in Fig. 7, exposure of hepatic Hep-G2 cancer cells to Y_2_O_3_ NPs at the IC50 concentration (13.15 µg/ml) resulted in a statistically significant (p < 0.001) increase in the expression level of apoptotic p53 and mitochondrial ND3 genes, along with marked decrease (p < 0.001) in the expression level of the anti-apoptotic Bcl2 gene compared to their expression level in untreated Hep-G2 cells.

## Discussion

Hepatic cancer is the second leading cause of cancer-related deaths globally. While treatments such as surgery, chemotherapy, and targeted therapies are available, they often fall short in effectively targeting liver tumors, highlighting the urgent need for more precise and efficient treatment modalities. As one of the most common and deadly cancers, hepatic cancer remains a critical area of research, particularly given the limited therapies currently available (Gosalia et al. 2017; Addissouky et al. 2024). This underscores the potential of nanomedicine, where nanoparticles like Y_2_O_3_ NPs could facilitate targeted drug delivery, enhance diagnostic imaging, or even provide direct therapeutic effects on liver cancer cells. However, the specific effects of Y_2_O_3_ NPs on hepatic cancer cells are not yet fully understood. Therefore, this study was undertaken to assess the impact of Y_2_O_3_ NPs on cell viability, genomic and mitochondrial DNA integrity, ROS generation, and apoptosis induction in Hep-G2 hepatic cancer cells.

Our SRB assay results highlighted the potential cytotoxicity of Y_2_O_3_ NPs towards hepatic Hep-G2 cancer cells, as evidenced by the marked, concentration-dependent reduction in cell viability and the remarkably low IC50 value of 13.15 µg /ml observed after 72 h of exposure to Y_2_O_3_ NPs. These findings are consistent with recent studies reporting a strong selective cytotoxic effect of Y_2_O_3_ NPs on human triple-negative MDA-MB-231 breast cancer cells and epidermoid A431 skin cancer cells (Emad et al. 2023; Mohamed et al. 2025).

The observed reduction in Hep-G2 cell viability is likely due to the disruption of essential cellular functions, potentially caused by excessive ROS generation. ROS are highly reactive molecules that can damage cellular macromolecules such as lipids, proteins, and DNA, disrupting the balance between the antioxidant defense system and oxidative agents. This imbalance leads to oxidative stress and various DNA lesions, including base oxidation and DNA strand breaks (Maynard et al. 2009; Safwat et al. 2022; Kumar et al. 2024b , 2024c). Over ROS production by Y_2_O_3_ NPs was manifested in this study by the marked elevation in the fluorescence intensity emitted by Y_2_O_3_ NPs-treated Hep-G2 cells compared to untreated cells. This finding further supports the role of ROS over-generation in the Y_2_O_3_ NPs cytotoxic effects observed in this study.

ROS are normally generated as byproducts of normal cellular processes, particularly in the mitochondria during oxidative phosphorylation. However, when ROS accumulate in excess, they can induce cytotoxicity and contribute to various pathological conditions. The cytotoxic effects of ROS are primarily mediated through a series of interconnected pathways that cause cellular damage and activate death signals. These pathways include DNA damage, mitochondrial dysfunction, inflammation, and disruption of the cellular redox balance, ultimately leading to genomic instability, impaired cellular function, and cell death. A key mechanism by which ROS induce cytotoxicity is through the damage to cellular DNA. ROS can cause various forms of DNA damage, including base modifications, strand breaks, and crosslinking, all of which can result in genomic instability and mutations (Emad et al. 2023; Mohamed et al. 2025; Yu et al. 2020).

Genomic instability is a hallmark of nanoparticle-induced toxicity, with the overproduction of ROS by nanoparticles being a major driver of this instability. DNA is particularly susceptible to ROS-mediated damage, and when ROS level exceeds baseline level, they exacerbate DNA damage, leading to mutations, chromosomal aberrations, and, most critically, the formation of single-strand breaks and double-strand breaks. These types of damage are critical contributors to the destabilization of the genome (Yu et al. 2020; Singh et al. 2022; Kumar et al. 2023). In this study, the exposure of Hep-G2 hepatic cancer cells to Y_2_O_3_ NPs at the IC50 concentration (13.15 µg/ml) led to dramatic genomic DNA damage, as manifested by the marked increase in both single- and double-stranded DNA breaks detected by the alkaline comet assay. This assay, known for its high sensitivity to DNA breaks, revealed a substantial disruption of the genomic integrity. The occurrence of these breaks resulted in considerable genomic instability and elevated ROS generation, likely driving the observed genomic instability in Hep-G2 cells. This highlights the critical role of oxidative stress in nanoparticle-induced cellular damage, which likely contributed to the cytotoxic effects of Y_2_O_3_ NPs in Hep-G2 cancer cells. These findings align with recent studies by Emad et al*. *(2023) and Mohamed et al. (2025), who also reported significant ROS overproduction and severe genomic DNA damage upon exposure to Y_2_O_3_ NPs in human triple negative MDA-MB-231 breast cancer and epidermoid skin A431 cancer cells.

In addition to genomic instability induction, excessive ROS generation can lead to mitochondrial dysfunction, which further exacerbates oxidative stress and promotes apoptosis. Mitochondria are the primary source of ROS in cells and are highly vulnerable to oxidative damage. Damage to mitochondrial DNA and disruption of mitochondrial function can impair cellular energy production, trigger the release of pro-apoptotic factors, and ultimately induce cell death (Kumar et al. 2024a; Kuo et al. 2022; Mohamed et al. 2023). In this study, Y_2_O_3_ NPs induced mitochondrial damage in Hep-G2 cells was evidenced by a marked reduction in fluorescence emitted by Y_2_O_3_ NPs-treated Hep-G2 cells compared to untreated cells. Therefore, the observed cytotoxicity in Hep-G2 cells exposed to Y_2_O_3_ NPs is likely attributed to combined effect of excessive ROS-induced genomic instability and mitochondrial dysfunction.

Furthermore, increased ROS level can activate various signaling pathways that promote cell death, including the intrinsic apoptotic pathway. This pathway, regulated by the mitochondria, activates stress-responsive mechanisms like the p53 pathway, which in turn activates pro-apoptotic genes and inhibits anti-apoptotic genes, further driving cell death in response to excessive oxidative damage (Villalpando-Rodriguez and Gibson 2021; An et al. 2024). Consequently, the excessive ROS generation induction by Y_2_O_3_ NPs demonstrated in Hep-G2 cells resulting in DNA damage, genomic instability, and mitochondrial dysfunction, ultimately stimulating cell death.

Apoptosis induction by Y_2_O_3_ NPs was confirmed in this study by the significant increases in the number of apoptotic and necrotic Hep-G2 cells, as detected by Flow cytometry following a 72-h exposure to Y_2_O_3_ NPs. This ability of Y_2_O_3_ NPs to induce apoptosis in Hep-G2 cells is a crucial finding, suggesting their potential for use in cancer therapy. Apoptosis, or programmed cell death, is a natural mechanism for eliminating damaged or dysfunctional cells. In cancer therapy, promoting apoptosis in tumor cells is an effective strategy to limit tumor growth and progression. Apoptosis and necrosis are interconnected, and under extreme stress conditions such as high ROS level, apoptotic cells can transition into necrosis. In this study, the presence of necrotic cells following Y_2_O_3_ NPs treatment is likely due to oxidative stress, mitochondrial dysfunction, and the inability of apoptotic cells to complete their programmed death efficiently (Peng et al. 2022; Mohamed et al. 2024; Kumar et al. 2024d). Our qRT-PCR analysis data show that the apoptosis observed in this study was likely a result of the activation and upregulation of the apoptotic p53 and mitochondrial ND3 gene expression, alongside significant inhibition and downregulation of the anti-apoptotic Bcl-2 gene expression in Hep-G2 cells.

In line with previous studies, the activation and upregulation of the apoptotic p53 and mitochondrial ND3 genes, along with the downregulation of the anti-apoptotic Bcl-2 gene, collectively trigger apoptosis in Hep-G2 hepatic cancer cells via the intrinsic mitochondrial pathway. p53 activation promotes the expression of pro-apoptotic genes, leading to mitochondrial dysfunction and the release of apoptotic factors, such as cytochrome c. The upregulation of mitochondrial ND3, linked to increased mitochondrial activity and ROS production, further exacerbates mitochondrial damage, amplifying apoptotic signaling. Concurrently, the downregulation of Bcl-2 removes its protective effect on the mitochondria, allowing for the activation of pro-apoptotic proteins like Bax. These combined molecular alterations destabilize the mitochondria, activate the caspase cascade, and ultimately lead to cell death, providing a potential mechanism for the cytotoxic effects of Y_2_O_3_ NPs in cancer therapy (Emad et al. 2023; Mohamed et al. 2025).

Although our findings demonstrated the potential therapeutic effect of Y_2_O_3_ NPs in hepatic cancer treatment through apoptosis induction, several notable limitations must be addressed. Thorough evaluation of their in vivo toxicity, immune response, long-term stability, optimal dosing, targeting efficiency, human clinical trials and manufacturing challenges is essential for Y_2_O_3_ NPs successful translation from preclinical studies to clinical application. A deeper exploration of these factors will provide a more comprehensive understanding of their feasibility, ensuring a safer and more effective use in cancer therapy.

## Conclusion

Collectively, the present study highlights the potential of Y_2_O_3_ NPs as a promising therapeutic strategy for hepatic cancer. Exposure to Y_2_O_3_ NPs induced a strong concentration-dependent reduction in in the viability of Hep-G2 hepatic cancer cells. This cytotoxic effect is likely due to the marked overproduction of ROS, which caused extensive DNA damage and a notable loss of mitochondrial membrane potential. Furthermore, treatment with Y_2_O_3_ NPs led to a significant increase in the expression of the pro-apoptotic p53 gene and mitochondrial ND3 gene, while significantly decreased the expression of the anti-apoptotic Bcl-2 gene, which likely contributes to the induction of apoptosis in Hep-G2 cells. While these results are promising, additional research is necessary to fully elucidate the mechanisms behind the therapeutic action of Y_2_O_3_ NPs and to assess their long-term effects and safety in vivo. These findings lay the groundwork for further exploration of Y_2_O_3_ NPs in nanomedicine and liver cancer treatment, though the limitations of this study warrant caution in making definitive therapeutic claims at this stage for this devastating disease.

## Data Availability

The datasets used and/or analyzed during the current study are available from the corresponding author on reasonable request.
